# Management of patients with reduced dihydropyrimidine dehydrogenase activity receiving combined 5-fluoruracil-/capecitabine-based chemoradiotherapy

**DOI:** 10.1007/s00066-024-02287-7

**Published:** 2024-09-04

**Authors:** E. Hoffmann, A. Toepell, A. Peter, S. Böke, C. De-Colle, M. Steinle, M. Niyazi, C. Gani

**Affiliations:** 1 University Hospital for Radiation Oncology and Radiotherapy, University Hospital Tübingen, Tübingen, Germany; 2 Department for Diagnostic Laboratory Medicine, Institute for Clinical Chemistry and Pathobiochemistry, University Hospital Tübingen, Tübingen, Germany; 3 Department for Radiation Oncology, Sacro Cuore Don Calabria Hospital, Negrar-Verona, Italy

**Keywords:** Rectal cancer, Multimodal treatment, Genetic variants, Personalized medicine, Pre-therapeutic testing

## Abstract

**Background:**

5‑Fluoruracil (5-FU) and its oral prodrug capecitabine are mainstays in combined chemoradiotherapy regimens. They are metabolized by dihydropyrimidine dehydrogenase (DPYD). Pathogenic variants of the *DPYD* gene cause a reduction in DPYD activity, leading to possibly severe toxicities. Therefore, patients receiving 5‑FU-/capecitabine-based chemoradiotherapy should be tested for *DPYD* variants. However, there are limited clinical data on treatment adjustments and tolerability in patients with decreased DPYP activity receiving combined chemoradiotherapy. Therefore, a retrospective analysis of the toxicity profiles of patients with decreased DPYD activity treated at our center was conducted.

**Materials and methods:**

For all patients receiving 5‑FU-/capecitabine-based chemo(radio)therapy at our department, DPYD activity was routinely tested. Genotyping of four *DPYD* variants (DPYD*2A, DPYD*13, c.2846A *>* *T*, and haplotype B3) was conducted according to the recommendation of the German Society for Hematooncology (DGHO) using TaqMan hydrolysis polymerase chain reaction (PCR; QuantStudy 3, Thermo FisherScientific, Darmstadt). *DPYD *variants and activity score as well as clinical data (tumor entity, treatment protocol, dose adjustments, and toxicity according to the Common Terminology Criteria for Adverse Events [CTCAE]) were assessed and reported.

**Results:**

Of 261 tested patients, 21 exhibited *DPYD *variants, 18 of whom received chemoradiotherapy. All but one patient was treated for rectal or anal carcinoma. The observed rate of *DPYD* variants was 8.0%, and heterozygous haplotype B3 was the most common (5.75%). One patient exhibited a homozygous *DPYD* variant. DPYD activity score was at least 0.5 in heterozygous patients; chemotherapy dose was adjusted accordingly, with an applied dose of 50–75%. CTCAE grade 2 skin toxicity (50%) and grade 3 leukopenia (33.3%) were most common. One patient experienced a transient grade 4 increase in transaminases. All high-grade toxicities were manageable with supportive treatment and transient. No CTCAE grade 5 toxicities related to 5‑FU administration were observed.

**Conclusion:**

With dose reduction in heterozygous patients, toxicity was within the range of patients without *DPYD* variants. Our clinical data suggest that dose-adapted 5‑FU-/capecitabine-chemoradiotherapy regimens can be safely considered in patients with heterozygous clinically relevant *DPYD* variants, but that the optimal dosage still needs to be determined to avoid both increased toxicity and undertreatment in a curative setting.

## Introduction

5-Fluoruracil (5-FU) and its oral prodrug capecitabine are mainstays in the treatment of many tumor entities including breast, head and neck, and gastrointestinal neoplasms. In radiotherapy, 5‑FU is a vital component of several treatment protocols, such as for rectal [1–3], anal [4], and bladder cancer [5], as it has been shown to have radiosensitizing effects [6].

Both 5‑FU and capecitabine are metabolized to 80% by dihydropyrimidine dehydrogenase (DPYD), which metabolizes 5‑FU quickly, leading to a half-life of about 8 to 20 min after bolus injection [7, 8]. However, several clinically relevant genetic *DPYD* variants that can lead to a reduction in DYPD activity and thus to increased 5‑FU serum levels have been identified. Of over 160 identified genetic variants of the *DPYD* gene, the following four clinically relevant variants are most common in Caucasians populations [9, 10] and therefore routinely tested for before treatment initiation at our hospital: DPYD*2A, DPYD*13, c.2846A *>* *T*, and haplotype B3.

Generally, 5‑FU-related side effects include gastrointestinal adverse events like mucositis and colitis and severe hematological side effects with pronounced anemia and leukopenia, as well as cardiotoxicity, neurotoxicity, and dermatological toxicities such as hand–foot syndrome [8, 11]. The risk of high-grade toxicity ranges from up to 5% for grade 4 and to 20–50% for grade 3 toxicities in patient populations without *DPYD* variants [12–15]. In up to 1%, toxicity can lead to lethal outcomes [13, 16]. As the probability of high-grade toxicity already ranges to up to 30% even without a reduction in DYPD activity, the presence of *DPYD* variants can cause a reduction in DPYD activity even in heterozygous patients, thereby leading to insufficient 5‑FU metabolization and increasing the risk for severe and possibly lethal toxicities [11, 12, 17].

Therefore, several national and international guidelines recommend that all patients scheduled for 5‑FU-/capecitabine-based chemotherapy should undergo phenotype or genotype testing prior to treatment initiation, so that chemotherapy doses can be adjusted accordingly [8, 9, 18, 19]. While toxicities and dose adjustments have been thoroughly studied for systemic therapy alone, there are limited clinical data on treatment protocol adjustments and tolerability in patients with reduced DPYD activity receiving combined chemoradiotherapy [21–23, 26]. Therefore, it is unclear whether recommendations regarding systemic therapy can readily be transferred to chemoradiotherapy treatment protocols or whether they might need to be adjusted for combined treatment. As 5‑FU and capecitabine have radiosensitizing properties, their application in a radiotherapy setting might lead to a further increase in toxicity. Conversely, as 5‑FU is a vital part of many curative chemoradiotherapy regimens, the elimination of 5‑FU from these protocols might prove to be a therapeutic disadvantage, resulting in an insufficient dose and compromising the chances of cure.

In this report, the incidence of* DPYD* variants in patients tested prior to chemo(radio)therapy at our department was assessed. Clinical characteristics, DPYD activity scores, treatment protocol and protocol adjustments, and toxicity profiles according to the Common Terminology Criteria for Adverse Events (CTCAE) in patients with reduced DPYD activity were analyzed.

## Materials and methods

All patients tested for *DPYD* variants prior to receiving 5‑FU-/capecitabine-based chemoradiotherapy at our department since January 2020 were eligible for analysis. Patients who received palliative or adjuvant chemotherapy without parallel radiotherapy were excluded from further analysis.

Genotyping of four *DPYD* variants (DPYD*2A, DPYD*13, c.2846A *>* *T*, and haplotype B3 [9, 10, 18]) was routinely conducted according to the recommendation of the German Society for Hematooncology (DGHO) [20] using TaqMan hydrolysis polymerase chain reaction (PCR; QuantStudy 3, Thermo FisherScientific, Darmstadt). *DPYD* variants and activity score were documented. Chemotherapy doses for treatment were adjusted according to the recommendations of the DGHO. 5‑FU serum levels were assessed after 24 h after the first chemotherapy application to enable further dose adjustments in case of increased serum levels for the following chemotherapy cycle. During treatment and for 4 weeks after completion of chemotherapy, weekly blood analysis was conducted (testing blood cell count, serum creatinine, and C‑reactive protein [CRP] at minimum). Likewise, weekly patient visits were conducted, during which treatment side effects and initiated treatments, if necessary, were documented.

For this analysis, the incidence of *DPYD *variants and DPYD activity scores for patients with clinically relevant variants were reported. Clinical and oncological data (tumor entity, treatment protocol, choice of chemotherapy agents, and chemotherapy adjustments) were analyzed. Toxicity profiles during treatment of patients with a DPYD alteration were assessed from documentation of weekly patient visits during treatment (regarding skin, hematological, neurological, cardiological, and mucosal toxicity as well as pathologic laboratory findings) according to the CTCAE [24].

This study was conducted in compliance with the Declaration of Helsinki. The study was approved by and registered with the ethics committee of the University of Tubingen (959/2021BO2). Broad consent to analyze clinical data and outcome was given. All patients agreed to genetic testing for assessing *DPYD* variants.

## Results

### Patients’ clinical and oncological characteristics

**Fig. 1:**
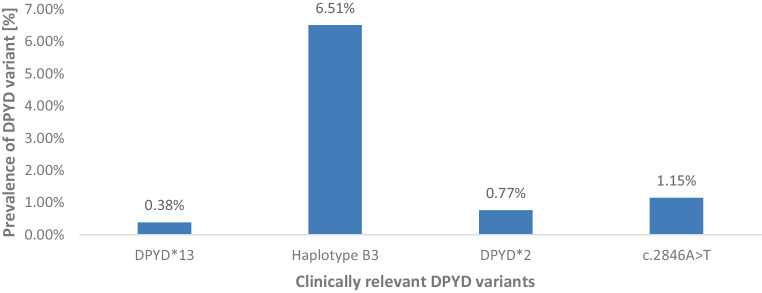
Distribution of clinically relevant *DPYD* variants in the screened cohort*. *Incidence reported among *n* = 261 screened patients (whole cohort). All but one patient with clinically relevant *DYPD* variants exhibited heterozygous variants. That patient, presenting with a homozygous haplotype B3, received no radiotherapy but only palliative chemotherapy without 5‑FU. Two other patients were also only treated with chemotherapy and thus excluded from further analyses. One patient who received combined chemoradiotherapy presented with both haplotype B3 and *c.2846 A > T* variants.

Between January 2020 and February 2024, 261 patients scheduled for 5‑FU- or capecitabine-based chemo(radio)therapy at our department were tested for four common and clinically relevant *DPYD* variants. *DPYD* variants were observed in 21 patients (8.0%). With one exception, all presented with a heterozygous genetic *DPYD* gene variation, among which haplotype B3 was most prevalent (*n* = 17/21 patients, 80.9%; distribution of variants detailed Fig. 1). One patient had both a heterozygous haplotype B3 and a *c.2846A* *>* *T* variant.

Three patients (one of whom was the only patient with homologous haplotype B3) received adjuvant or palliative chemotherapy without radiation and were thus excluded from further analysis. The tumor entities of patients receiving chemoradiotherapy included predominantly lower gastrointestinal neoplasms (rectal cancer, *n* = 11, and anal cancer, *n* = 6). In one case, a patient presented with both anal carcinoma and leiomyosarcoma of the uterus, for which she received operative therapy once chemoradiotherapy for anal carcinoma had been completed. Further patient characteristics are detailed in Table 1. All patients were treated with intensity-modulated radiotherapy for optimal organ at risk sparing [25].

**Table 1:** Patient characteristics

**Gender distribution male vs. female**	8 vs. 10 (44.4% vs. 55.6%)
**Age at start of treatment (years)**	63.0; SD 9.9
**Tumor entity**	
*Rectal cancer*	11 (61.1%)
*Anal cancer*^*a*^	6 (33.3%)
*Oral squamous cell carcinoma*	1 (5.6%)
*Uterine leiomyosarcoma*^*a*^	1 (5.6%)
**T**	
*1*	3 (16.7%)
*2*	3 (16.7%)
*3*	10 (55.6%)
*4*	2 (11.1%)
**N**	
*Negative*	7 (38.9%)
*Positive*	11 (61.1%)
**M**	
*Negative*	17 (94.4%)
*Positive*^*b*^	1 (5.6%)

Detailed in absolute number of patients (percentage detailed in brackets)

^a^One patient presented with both anal cancer and uterine leiomyosarcoma. The leiomyosarcoma was encompassed in the radiation field and resected following radiotherapy

^b^In one patient with anal cancer, a single osseous lesion in the first sacral vertebra was suspected on imaging. The osseous lesion received a simultaneous integrated boost of 58.8 Gy

Sixteen patients received dose-adapted 5‑FU (dose reduction of 50–75%; Table 2). No chemotherapy dose had to be reduced in response to 5‑FU serum levels determined 24 h after the first 5‑FU application. Based on genetic findings, physicians’ assessment, and patients’ wishes, 5‑FU was omitted from chemotherapy regimens in 2 cases (activity score 0.5 and 1.5, respectively), with alternatives applied (mitomycin after one 24-hour 5‑FU infusion after which the patient asked for 5‑FU discontinuation and cisplatin), so that in total, 16/18 (88.9%) patients received 5‑FU-based therapy regimens. Further radiotherapy and chemotherapy details are listed in Table 2. All patients completed radiotherapy treatment as planned without toxicity-related interruptions.

**Table 2:** DPYD score and treatment characteristics

*DPYD activity score*	
0.5	1 (5.6%)
1.0	3 (16.7%)
1.5	14 (77.8%)
*Treatment modification*	
Other chemotherapy (cisplatin, MMC)	2 (11.1%)
50% dose reduction	3 (16.7%)
75% dose reduction	13 (72.2%)
*Chemotherapy protocol*	
5‑FU or capecitabine/MMC^a^	6 [5] (33.3%, [27.8%])
5‑FU or capecitabine mono	8 (44.4%)
FOLFOX	3 (16.7%)
Cisplatin weekly (40 mg/m^2^)	1 (5.6%)
5‑FU^a^ vs. capecitabine	14 [13] vs. 3 (77.8%, [72.2%] vs. 16.7%)
*Radiotherapy protocol*	
50.4 Gy in 1.8 Gy per fraction +/− integrated or sequential boost^b^	15 (83.3%)
25 Gy in 5.0 Gy per fraction	2 (11.1%)
65.6 Gy in 2.05 Gy per fraction	1 (5.6%)

Detailed in absolute number of patients (percentage detailed in brackets). Number of patients without the one patient for whom 5-FU was stopped after one day at the request by the patient (detailed in square brackets)

*MMC* Mitomycin C, *5-FU* 5-fluorouracil, *FOLFOX* folinic acid, 5-FU, and Oxaliplatin

^a^One patient started combined 5‑FU/MMC chemotherapy. 5‑FU infusions were stopped after day 1 at request by the patient

^b^Boosts were administered both as simultaneous integrated boost and sequential boost to the primary tumor, pathologic lymph nodes, and, in one case, a single suspected metastatic osseous lesion. Total doses ranged from 54.0 to 65.6 Gy

### Side effects and toxicity profiles

**Fig. 2:**
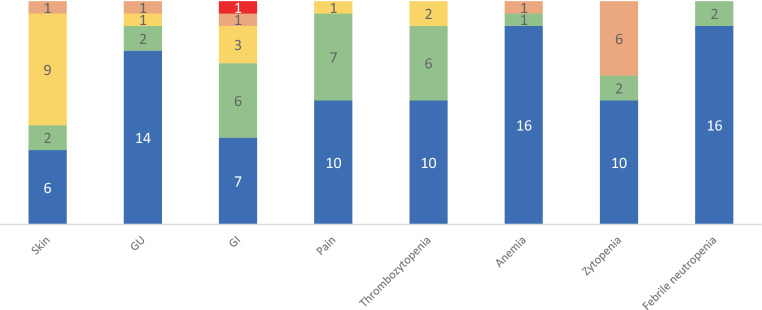
Observed toxicities according to CTCAE criteria. *N* = 18 patients. *Blue* no toxicity, *green* grade 1, *yellow* grade 2, *orange* grade 3, *red *grade 4. *GU* genitourinary (dysuria, urinary frequency), *GI *gastrointestinal (diarrhea, rectal bleeding). The grade 4 toxicity observed was a significant but transient increase in transaminases after the first chemotherapy application, which resolved spontaneously. Skin toxicity grade 1: faint erythema or dry desquamation; grade 2: moderate erythema and/or edema, patchy moist desquamation; grade 3: confluent moist desquamation, bleeding in response to minor trauma. Thrombocytopenia grade 1: > 75,000/ml; grade 2: 50,000–75,000/ml; grade 3: 25,000–50,000/ml. Anemia grade 1: hemoglobin < 10 mg/dl; grade 2: hemoglobin > 8 but < 10 mg/dl; grade 3: hemoglobin < 8 mg/dl, transfusion indicated. Cytopenia grade 1: corresponding to a decrease in blood cell count to 75% of initial cell count; grade 2: corresponding to a decrease in blood cell count to 50–75% of initial cell count; grade 3: corresponding to a decrease in blood cell count to 25–50% of initial cell count

Toxicity profiles of the analyzed patient cohort are detailed in Fig. 2. In 10 patients (55.6%), no toxicity higher than CTCAE grade 2 was observed. Of these patients, five (27.8%) suffered from grade 2 skin toxicity and one from grade 2 thrombocythemia, with otherwise no or only grade 1 toxicity reported.

Seven patients (38.9%) suffered grade 3 toxicities, mostly hematological. One patient treated for anal carcinoma experienced grade 3 desquamation without other high-grade toxicities. In six cases (33.3%), grade 3 cytopenia occurred during chemotherapy treatment. Four of these patients had additionally received mitomycin (treatment for anal carcinoma) and one received a FOLFOX regimen (5-FU, oxaliplatin, and folinic acid after completion of radiotherapy) as intensified chemotherapy for rectal cancer. Of these, one patient only received one 24-hour infusion of 5‑FU (absolute dose 1372.5 mg) before 5‑FU was discontinued based on the patient’s wishes. The patient had, however, received the full dose of mitomycin C (absolute dose 27.45 mg). In two cases with grade 3 cytopenia, patients received antibiotic treatment for febrile neutropenia. One patient suffered from a urinary tract infection while in the other patient, no infection focus could be determined. Both patients improved under antibiotic treatment. Treatment could be completed according to plan. Only one patient with grade 3 cytopenia received 5‑FU alone. This patient (*c.2846A* *>* *T* variant, rectal carcinoma) had other high-grade side effects with grade 2 pain; grade 3 mucositis, diarrhea, and pre-renal kidney failure; and an afebrile urinary tract infection due to reduction in fluid uptake due to painful mucositis. The patient received supportive treatment (pain medication, intravenous fluids) and was able to complete the second course of 5‑FU chemotherapy in 50% dose reduction as planned.

One patient suffered a rectovaginal fistula after treatment for anal carcinoma. However, in initial staging, a mucosal infiltration of the vagina and thus a T4 tumor stage in imaging and clinical examination prior to treatment initiation was already present. The patient subsequently received operative therapy and a colostomy.

Only one grade 4 (5.5%) toxicity was reported. In a patient treated with two concomitant cycles of 5‑FU application for rectal carcinoma and a DPYD activity score of 1.0, a significant increase of transaminases was observed after the first cycle of chemotherapy, without any other high-grade toxicity. Hepatological consultation yielded no clear cause of the transaminase increase, and liver ultrasound was without pathological findings. As a relevant comorbidity and competing possible cause, rheumatological arthritis was reported. The transaminase increase resolved without further treatment and did not occur following the second cycle of 5‑FU or during adjuvant capecitabine treatment, so that a definite causal correlation with 5‑FU application remained unclear.

No CTCAE grade 5 toxicities occurred in response to 5‑FU administration.

## Discussion

In our analysis, we report toxicity outcomes of patients with DPYD deficiency receiving combined chemoradiotherapy.

We found that overall, toxicity profiles were within the range reported for patients without DYPD deficiency. Most patients (55.6%) experienced no or only mild treatment side effects (CTCAE 1 or 2). Seven patients (38.9%) experienced grade 3 toxicity—mostly hematological—and one grade 4 transaminase increase was observed (5.6%), in line with the reported risk of 25–50% and 3–5.0%, respectively, of higher-grade toxicities in non-variant cohorts [12, 13]. There was no clear correlation between high-grade toxicity and the individual *DPYD* variant or activity score of the patients. In our cohort, no grade 5 toxicities were observed. Grade 3 and 4 toxicities were manageable and transient.

The incidence of CTCAE grade 2 and 3 skin toxicity (55.6%) in our analysis might also be related to the high number of patients with anal cancer and distal rectal cancer. Due to the proximity of the tumor to the skin in these cases and thus comparably high doses in the epidermis, (confluent) desquamation is more common in the radiotherapy of this tumor entity as compared to, e.g., proximal rectal cancer.

Apart from receiving other chemotherapy agents in addition to 5‑FU, the rate of grade 3 cytopenia might also be influenced by the radiotherapy treatment received. In the radiotherapy treatment of prostate, anal, and rectal cancer, pelvic lymph nodes are included in the adjuvant treatment volume, resulting in a significant dose to the iliac vessels and thus white blood cells. Several studies have shown that the irradiation of a significant blood volume can contribute to white blood cell changes [28–30], which might also have contributed to the observed cytopenia in our study.

Several guidelines have published a recommendation on *DPYD* variant testing and dose adjustments for systemic therapy. Dutch guidelines recommend genotyping of the abovementioned four *DPYD *variants prior to chemotherapy initiation. For an activity score of 0, 5‑FU/capecitabine should be avoided, and for an activity score between 0.5 and 1.5, chemotherapy should be started with a 50% dose reduction [31]—a higher dose reduction than recommended by German guidelines (50% reduction for an activity score of 1.0 and 25% reduction in the case of an activity score of 1.5 [20]). The European Medicines Agency (EMA) recommends either geno- or phenotyping (testing for uracilemia) prior to chemotherapy application and advises drug monitoring during treatment. It furthermore points out that dose adjustments according to genotyping have been validated as compared to testing for uracilemia and cautions that recommendations regarding genotyping for the abovementioned four *DPYD* variants only apply to Caucasian populations [8].

These dose adjustment recommendations have so far been applied analogously to the situation of patients receiving combined chemoradiotherapy. However, there are limited data available on the side effects of combination treatment in this group of patients. One large retrospective study on toxicity in patients undergoing combined chemoradiotherapy for mostly rectal cancer (71.7%) with 5‑FU as a single agent reported an increased risk for high-grade hematological and gastrointestinal toxicities in patients with *DPYD* variants who did not receive dose-adjusted chemotherapy regimens [21]. Of 828 patients, 34 *DPYD* variant carriers did not receive dose-adjusted chemotherapy in contrast to 22 patients whose chemotherapy dose was adjusted following *DYPD* testing. Patients who were treated with a reduced 5‑FU dose showed no increased risk for grade 3 or higher gastrointestinal toxicities such as diarrhea, nausea, and vomiting (9.1% dose-adjusted, not significant, vs. 17.6% not adjusted, *p* = 0.045, vs. 8% wild type), but still had a higher incidence of grade 3 or higher hematological toxicities than patients without *DYPD* variants (9.1% dose-adjusted, *p* = 0.083, vs. 11.8% not adjusted, *p* = 0.015, vs. 2.9% wildtype). The rate of mucositis was not increased. In our analysis, we found a markedly higher incidence of grade 3 cytopenia of 33.3%. However, contrary to the analysis by Lunenburg et al., 5 out of 6 patients in our study received a combination of 5‑FU and MMC, which is known to cause leukopenia [27], or an intensified regime with FOLFOX.

Saif et al. [22] retrospectively analyzed 21 patients treated for anal carcinoma using a combination of 5‑FU and MMC. In this study, patients who developed grade 3 toxicities during chemoradiotherapy were tested for *DYPD* variants. 5‑FU doses were adjusted accordingly, or a different chemotherapy agent was chosen after occurrence of a grade 3 toxicity and subsequent testing; subsequently, all patients completed combined treatment with no higher than grade 2 toxicity. The authors report an incidence in grade 3 or higher diarrhea of 38%, whereas in our cohort, only one case of grade 3 diarrhea (5.5%) was reported. Grade 3 anemia occurred in 38% of patients as compared to 1 patient in our study (5.5%), and almost half of the patients (47%) developed grade 3 or higher neutropenia in comparison to one third of patients in our analysis. This is presumably due to the fact that testing and thus dose reduction were only conducted if a grade 3 toxicity had already occurred, underscoring the need for pretherapeutic testing.

In a study focused on carboplatin/5-FU as a chemotherapy alternative to cisplatin in primary head and neck squamous carcinoma radiotherapy, Hanemaaijer et al. found a higher grade 3 and 4 toxicity rate regarding thrombocytopenia and leukocytopenia as well as a higher risk for treatment discontinuation in the carboplatin/5-FU cohort. However, no testing for *DYPD* variants was conducted [23]. Desilets et al. noted a reduction in the incidence of grade 3 toxicities—namely dysphagia, mucositis, and dermatitis—in patients receiving chemoradiotherapy for head and neck malignancies from 71% to 62% after the introduction of pretherapeutic genotyping for *DPYD* variants [32].

As in our retrospective analysis, none of the abovementioned studies reported a death related to treatment-associated toxicity during or after chemoradiotherapy, neither in dose-adjusted nor in non-adjusted regimens.

The incidence of toxicities in our analysis was not increased compared to patients without DPYD variants, in line with the limited published data on toxicity profiles in this group of patients. This begs the question of whether dose reduction in the chemoradiotherapy setting should be conducted analogously to systemic therapies or whether less reduction of 5‑FU would also not result in significantly increased toxicities. This question cannot be answered by our analysis but is important to avoid undertreatment by chemotherapy reduction in these patients.

## Limitations

This analysis focused on patients who were tested for the four most common *DPYD* variants. However, as high-grade toxicities are also observed in patients without heterozygous or homozygous DPYD variants, it is possible and likely that other variants carrying a risk for increased toxicity have not yet been identified. Also, different variants might not bear the same risk for high-grade toxicities. This underscores the need for publication of clinical data in order to identify patient populations with increased toxicity profiles.

The patient cohort analyzed comprised a diverse group regarding tumor entity and treatment protocol. Also, the absolute number of patients exhibiting a clinically relevant* DPYD* variant was—although within the reported prevalence in a Caucasian population—relatively small. Therefore, the analyzed group is too heterogenous to derive general recommendations for treatment adjustments and toxicity.

However, to our knowledge, only three other publications have so far focused on tolerability and the toxicity profile in patients with DPYD deficiency receiving 5‑FU in a combined chemoradiotherapy regimen [21–23]. Our analysis therefore contributes important clinical data for helping assess the safety of combined treatment in this patient cohort.

## Conclusion

In our cohort, the observed rate of *DPYD* variants of 8.0% was in accordance with reported rates in Caucasian populations (9%). The heterozygous haplotype B3 variant was most common. DPYD activity score was at least 0.5 in heterozygous patients, corresponding to residual DPYD activity. CTCAE grade 2 and 3 skin toxicity and CTCAE grade 3 leukopenia were the most commonly observed side effects. One grade 4 increase in transaminases resolved spontaneously and did not reoccur during following 5‑FU application. In patients receiving dose-adapted chemotherapy regimens, no CTCAE grade 5 toxicities were observed. Our clinical data suggest that dose-adapted 5‑FU/capecitabine chemoradiotherapy regimens in accordance with guidelines for systemic treatment can be considered in patients with heterozygous clinically relevant *DPYD* variants and an activity score of at least 0.5 without an increase in the risk of high-grade toxicity compared to non-variant patient cohorts. However, as data on patients with *DPYD* variants receiving combined chemoradiotherapy remain scarce, treatment adaptations and toxicity profiles should be reported in larger cohorts to assess the optimal dose of combined 5‑FU-based chemoradiotherapy protocols in this group of patients.
